# Comparison of Individual Sensors in the Electronic Nose for Stress Detection in Forest Stands

**DOI:** 10.3390/s23042001

**Published:** 2023-02-10

**Authors:** Tereza Hüttnerová, Sebastian Paczkowski, Tarek Neubert, Anna Jirošová, Peter Surový

**Affiliations:** 1 Faculty of Forestry and Wood Science, Czech University of Life Sciences (CZU Prague), Kamýcká 129, 165 21 Prague, Czech Republic; 2 Department of Forest Work Science and Engineering, Georg August University Göttingen, Büsgenweg 4, 37077 Göttingen, Germany

**Keywords:** unmanned aerial vehicles, electronic nose, 3D odor mapping, natural disturbance, early detection

## Abstract

Forests are increasingly exposed to natural disturbances, including drought, wildfires, pest outbreaks, and windthrow events. Due to prolonged droughts in the last years in Europe, European forest stands significantly lost vitality, and their health condition deteriorated, leading to high mortality rates, especially, but not limited to, Norway spruce. This phenomenon is growing, and new regions are being affected; thus, it is necessary to identify stress in the early stages when actions can be taken to protect the forest and living trees. Current detection methods are based on field walks by forest workers or deploying remote sensing methods for coverage of the larger territory. These methods are based on changes in spectral reflectance that can detect attacks only at an advanced stage after the significant changes in the canopy. An innovative approach appears to be a method based on odor mapping, specifically detecting chemical substances which are present in the forest stands and indicate triggering of constitutive defense of stressed trees. The bark beetle attacking a tree, for example, produces a several times higher amount of defense-related volatile organic compounds. At the same time, the bark beetle has an aggregation pheromone to attract conspecifics to overcome the tree defense by mass attack. These substances can be detected using conventional chemical methods (solid-phase microextraction fibers and cartridges), and it is proven that they are detectable by dogs. The disadvantage of classic chemical analysis methods is the long sampling time in the forest, and at the same time, the results must be analyzed in the laboratory using a gas chromatograph. A potential alternative novel device appears to be an electronic nose, which is designed to detect chemical substances online (for example, dangerous gas leaks or measure concentrations above landfills, volcanic activity, etc.). We tested the possibility of early-stage stress detection in the forest stands using an electronic nose Sniffer4D and compared the individual sensors in it for detecting the presence of attacked and dead trees. Our results indicate the promising applicability of the electronic nose for stress mapping in the forest ecosystem, and more data collection could prove this approach.

## 1. Introduction

Monitoring stress in a forest is an important component of forest management, and the early detection of stress conditions in forest stands can prevent significant economic and environmental losses. Forest stands are exposed as all living systems to stress factors, which can be of two origins: (1) internal (by physiological growth and development) and (2) external (by natural disturbances) [1]. The natural disturbance disorders interact both with each other (biotic x abiotic) but also with other factors such as increasing carbon dioxide in the atmosphere and climate change—increased temperature and drought [2,3,4,5]. The most vulnerable losses are brought mainly by biotic disturbances, mainly outbreaks of insects. The resistance to stress of a forest stand depends on its vitality and the degree and frequency of attack by insects [6]. The biggest damage is caused by insects feeding on the bast fibers of living trees, such as bark beetles [7]. The more extensive spread of bark beetles is mainly due to increasing temperature and drought [8,9]. The intensity and extent of the attack depend on direct and indirect interactions of the bark beetle with climate, susceptibility of forest, windthrow, and fires [10]. Increased temperature over the winter reduces bark beetle mortality and thus contributes to more generations per year [6].

To avoid economic and ecological losses, timely identification of forest stand stress is essential. Detection of stress in the forest is usually performed by a ground walk by trained specialists who can identify signs of stress. For the larger areas usually, remote sensing images are used to study the change in reflectance or values of some dedicated vegetation indices. The limiting factors of images obtained from a satellite are their temporal and spatial resolution; when mapping the spread of wildfire or insect outbreaks, these data are insufficiently accurate [11]. The solution might be the use of unmanned aerial vehicles (UAV), which can capture a local area with high spatial accuracy (in cm) [12]. UAV mapping is an important component in effective data collection, accurate terrain mapping, and measurement of toxic substances in the air. The UAV can be equipped with different types of sensors [13,14].

In the visible spectrum and near the near-infrared channel, the deteriorated state of forest stands can be monitored through vegetation indices (NDVI, Greenness Index). The accuracy of these indices is usually linearly correlated with the increasing length of the attack and intensity of the attack [15]. However, in some cases, an attacked tree does not show a change in spectral reflectance and is not marked as attacked/damaged in this approach [16]. A thermal camera was also used to capture the degraded state of forest stands. The appropriate timing of data collection is important. No significant disease-dependent correlation has yet been captured with morning collection, while the strongest relationship was obtained at the time of highest solar radiation, which coincides with the time of maximum photosynthetic activities near the trees, i.e., at noon [17].

Current rapid technological development brought into question studies related to chemical ecology, the studies which are focused on chemical processes such as the use of pheromones for beetle communication, the plant-beetle-based communication by attractants, and anti-attractants. Finally, it was proved that not only beetles can detect chemical substances, but dogs can be trained to do so [18]. This work ultimately proves it is possible to detect stressed trees by chemical substances. 

Relatively new sensors have appeared on the market based on microchip architecture and electronic noses that can convert the concentration of chemical substances into a voltage and then to a digital number. This sensor is used in the field of security and the military. In agriculture, these sensors have been used to detect odors from livestock, hazardous chemical, biological compounds, and for early detection and prediction of pest insects (for example: Aphid Infestation, Lepidoptera) [19,20]. In forestry, it is possible with these sensors to detect VOCs and other chemical components, which can identify stress factors [12,21]. Other sensors include H_2_S, HCL, nitrogen, and next SO_2_, CO, O_3_, NO_2_, and, for example, sensors for measuring the content of dust particles in the air (PM_1.0_, PM_2.5_, PM_10_). Remote sensing methods currently seek to obtain chemical data, and the electronic nose could be potentially suitable for calibration ground measurement [22].

**Figure 1:**
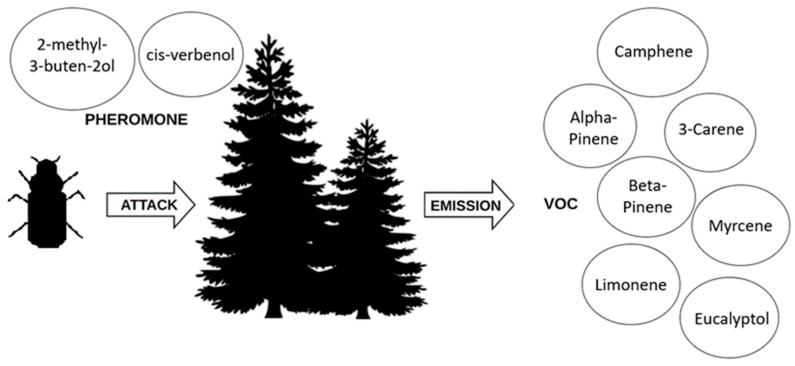
A simplified scheme—when a bark beetle attacks, trees secrete an increased amount of volatile organic substances; on the other hand, the male nesting in the tree starts to produce an aggregation pheromone from his body to attract females to mate.

During the attack, the pioneer beetles who search for appropriate habitats are the freshly emerged males. Once they land on the target host tree and successfully start to bore in the bark, they start producing aggregation pheromone (2-methyl-3-buten-2-ol and cis-verbenol) to attract conspecifics for a mass attack (Figure 1) [23,24]. Eco-friendly strategies to monitor pest insects have received huge attention due to reducing pesticide usage. The pheromones and VOCs act as powerful tools against pest insects [25,26]. Plants may respond to an insect attack with the emission of VOCs, and these odors may be correlated with the early attack [25,26]. Interestingly, plants may differentiate in their response between mechanical and herbivore damages, such as those caused by pest insects [27,28].

Elevated levels of VOCs can be detected by conventional chemical methods, cartridges, or solid-phase microextraction fibers (SPME) [29,30]. The increased emissions from infested trees were 6–20 times higher than healthy individuals [30,31]. Measurements using cartridges and solid-phase microextraction fibers provide only single-point measurements; neither fibers nor cartridges can be used repeatedly in the field. Proven chemical methods yield accurate measurements, but the data must first be analyzed on a gas chromatograph. During SPME data collection, fibers are exposed to air, typically for one hour. In open space, this time could increase even more. Gas chromatography analysis of one SPME or cartridge takes about 10–30 min, depending on the method used [31,32]. These steps delay the final statistical evaluation and eventual remediation of the attacked trees.

In this research, we hypothesized that there is a substance detectable by the electronic nose which indicates the presence or near distance of attacked and/or dead trees. We hypothesized that from the set of sensors included in the electronic nose, some or all would react as the distance to the dead and attacked tree becomes smaller, indicating the higher concentration of the substance present in the stressed tree. Our research aims to verify the applicability of the electronic nose for stress mapping in the forest environment. Our goal is to find suitable sensors that will respond to the presence of a substance identifying infested trees [33,34].

The electronic nose appears as an important tool to detect VOCs in different scenarios, including the emission of VOCs caused by herbivory-induced species of pest insects. This research could be a valuable part of new strategies against an outbreak of pest insects such as bark beetles. The benefit of this study is the comparison of individual sensors of the electronic nose for detecting stress in the forest ecosystem. Thus, we bring pilot research for scientists to determine which sensors are more appropriate to choose for analyzing the occurrence of bark beetle attacks in the following season. Timely identification would reduce environmental and economic damage.

In light of the bark beetle outbreaks and the current detection method, which can detect stress in forests in the late stage of infestation, the present brief report sets out to examine the following:

1. Is the specific substance that identifies stressed trees detectable by the Sniffer4D electronic nose?
2. What sensors will correlate with distance from stressed trees?
3. Can stress be detected by these sensors even above treetops using UAVs where the concentration is hypothetically lower?

## 2. Materials and Methods

### 2.1. Study Site

**Figure 2:**
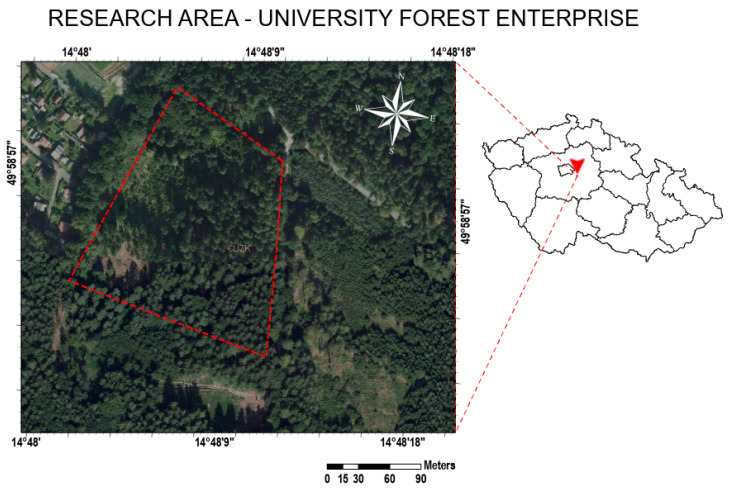
Location of study plot in Vyžlovka, Czech Republic, in a coordinate system S-JTSK Krovak East North, data source: ČÚZK.

The study was conducted at the property of the University Forest Enterprise near Vyžlovka, southeast of Prague (Figure 2). The forest is used for forestry research and teaching, and the particular forest stand is a single-story with a density of stocking 0.8. Field experiments were established in a mixed stand, where the dominant stand is 80–100-year-old allochthonous Norway spruce with accessory species beech, oak, and maple [35]. The terrain is relatively flat, and the type of soil is brown soil, strongly acidic podzolized. The primary rock in the area is slightly (to medium) grained two-mica granite [36]. The study region is a slightly warm area characterized by an average yearly temperature of 8 °C and an average precipitation of 600 mm [37].

### 2.2. Data Collection

**Figure 3:**
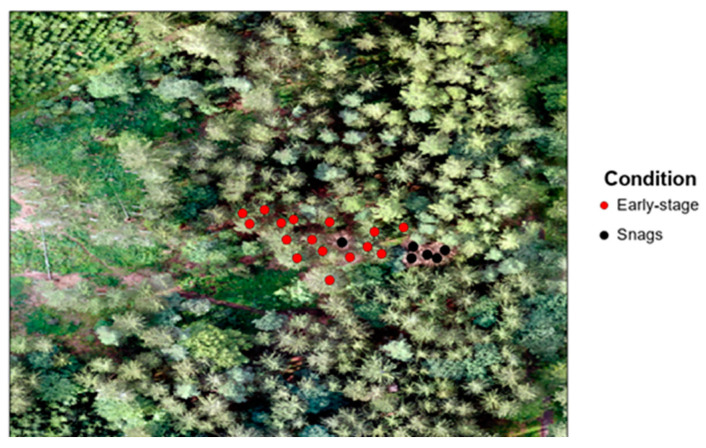
The orthomosaic was created from images taken from the UAV DJI Mavic with a visible spectrum in channel R, G, B. The image shows a point layer of trees; black points represent snags, red points indicate an early-stage infestation of trees, on the left is a clearing area; the other trees were not attacked.

We collected data on 24 August 2022; ground collection took place from 12:10 to 12:25 and 16:00 to 16:15, and collection using UAV took place between 15:40 and 15:55. On the ground level, we recorded during data collection a temperature of 20–23 °C and a humidity of 38.8–52.0%; during flight, we recorded 19–20 °C and a humidity of 45–50%. Data collection took place at two heights, ground measurement and above the canopy. The primary statistical description of the collected data is in Table 1, and the kernel density figures of individual sensors are given in Appendix A. Before the actual measurement, the stand was visually evaluated, and each tree’s health status and degree of infestation were examined (Figure 3). The state of the infestation was evaluated by a field survey, during which we evaluated the visual condition of the bark and needles and the development phase of the bark beetle. A tree that had not yet spawned the next generation was identified as the early stage of infestation. Trees that already had visible outcomes, holes, and needles that changed spectral expression were identified as dead trees. This is a classic forestry management tool for the identification of an infested tree in the Czech Republic.

**Figure 4:**
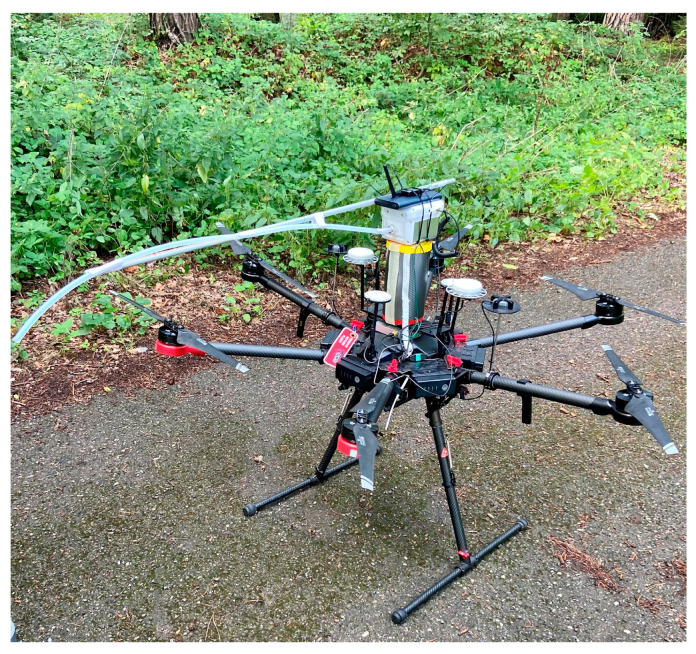
Electronic nose Sniffer 4D placed on multi-copter DJI Matrice 600; Teflon meter tube was used for sampling air.

**Figure 5:**
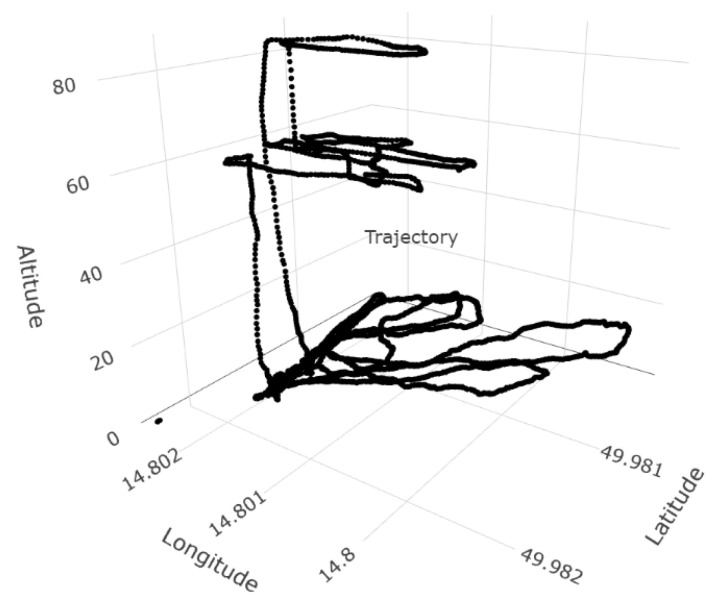
The trajectory of the sensor’s measurements. The measurement was first carried out on the ground level and then by an unmanned aerial vehicle—first; we descended just above the treetops (approx. 60 m), then we made a short flight at the height of 80 m.

Data were collected under the canopy by carrying the sensor and above the tree canopy by flying the DJI Matric 600 Pro multi-copter (©2022 SZ DJI Technology Co., Ltd.: Shenzhen, China). The electronic nose was placed on the drone, and air collection took place through a sampling tube, which was placed one meter in front of the drone to minimize the multi-copter’s downwash effect (Figure 4). The flight was carried out manually due to numerous obstacles during the flight, mainly under the crowns of trees. Large horizontal and vertical gaps were kept in flight (Figure 5). The first flight was carried out as low as possible above the crowns (60 m), and then we flew 20 m higher to verify the captured VOC substances at a greater distance from the attacked trees.

### 2.3. Electronic Nose

The Sniffer 4D V2 sensor (©2022 SZ Soarability Technologies Co., Ltd.: Shenzhen, China) is a multi-gas and mapping device used in drone and ground measurement applications. The sensor is composed of multi-gas detection hardware and analysis software. This system is capable of measuring and, at the same time, visually presenting the detected concentrations of gases of interest in real-time. The sensor is constructed from a 1 GHz ARM main processing unit (processing chip) and 512 MB memory. The sensor can measure temperature, humidity, and pressure (range −40~85 °C, 0~100% RH, 30 kPa~110 kPa; theoretical resolution: 0.1 °C, 0.1% RH, 0.01 kPa; time resolution: 1 s. The sensor was equipped with components for measuring VOCs, SO_2_, CO, O_3_ + NO_2_, PM_1.0_, PM_2.5_, PM_10_, CxHy, H_2_S, and HCL [38]. As part of the measurement, the response of all the sensor components was evaluated, aiming to find the specific sensor or sensors that would be the most sensitive to the chemical substances released during a bark beetle attack.

### 2.4. Analysis of Sensors Data

**Figure 6:**
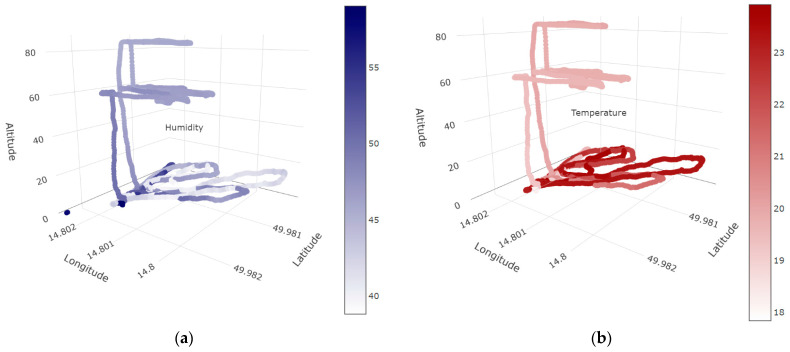
3D models present the humidity (**a**) and temperature (**b**) measured by the sensor during the entire field experiment.

The measurement values for individual sensors were evaluated using Pearson’s correlation coefficient to determine the correlation between the distance from the trees and the value measured by the sensor. The assumption is that these values depend on each other; the closer we are to early-stage infestation trees/dead trees, the higher the values are measured by the sensors, thus representing a negative correlation coefficient. The weather affects the bark beetles’ activity, so measuring temperature and humidity is also important. The ideal temperature is around 25 °C without rain occurrence on data collection day. The 3D models represent humidity (Figure 6a) and temperature (Figure 6b) measurements during the entire data collection. Temperature and humidity significantly affect the spread of bark beetles; for example, the bark beetle is active at a temperature of 16 to 32 °C, and the optimum flight temperature is 22–26 °C (for searching for suitable host trees). In laboratory conditions, a relative humidity of 70% is used for growing bark beetles [39,40].

## 3. Results

The individual sensors of the electronic nose are cross-sensitive, i.e., a sensor primarily created for CO_2_ detection may be sensitive to specific compounds of VOCs or pheromones. Therefore, in our study, we verified several sensors of the electronic nose, intending to find the most responsive ones to the presence of dead trees and trees attacked by the bark beetle. Such an application is in the initiation phase, and more experiments are needed. In this work, we evaluated the responsiveness of the individual sensors for indicating the distance of dead trees and trees attacked by a bark beetle. According to the study questions, we find out the following: (1) the results point to the potential use of the electronic nose for stress monitoring in forest stands and (2) individual sensors can detect specific compounds that identify stress. The highest sensor values were recorded near the most attacked trees. The highest correlation was recorded with the HCL, SO_2_, and H_2_S sensors during ground measurements. The ability to detect stress above the canopy using a UAV has not been proven. A more detailed explanation of the results and their causes is below.

Table 2 presents the Pearson correlation coefficient results for the ground first and second measurements for dead trees. At the first measurement, the highest correlation was recorded for the HCL (−0.456) sensor, followed by SO_2_ (−0.209) and H_2_S (−0.132). During the second ground measurement, the most accurate measurement coefficient values were for the H_2_S (−0.432) sensor, next for HCL (−0.378) and SO_2_ (−0.360). We successfully managed to detect a chemical substance that identified the presence of dead trees, both during the first ground measurement and during the second one.

Table 3 presents Pearson correlation coefficients for the first and second ground measurements for early-stage infestation trees. This data collection also fulfilled the assumption that any of the sensors would react to substances that identify the presence or appear near the location of the early-stage infestation trees. At the first measurement, the highest accuracy was recorded for the HCL (−0.427) sensor, followed by SO_2_ (−0.164) and H_2_S (−0.100). During the second ground measurement, the most accurate measurement coefficient values were for the H_2_S (−0.486) sensor, next for SO_2_ (−0.480), and C_x_H_y_ (−0.339).

Table 4 presents the Pearson correlation coefficient results for the UAV measurements for early-stage infestation and dead trees. No higher correlation coefficient values were achieved with UAV data collection. The reason for these results may lie in the lower concentrations above the forest canopy of the substances released during the attack. Another hypothetic cause could have been weather conditions (e.g., the wind taking the substances away, though no significant wind was registered on the day); in the future, it would be advisable to equip the electronic nose with an anemometer for the next measurements. As part of measurements with an anemometer, we will be able better interpret and record the spread of chemical compounds in the forest environment.

**Figure 7:**
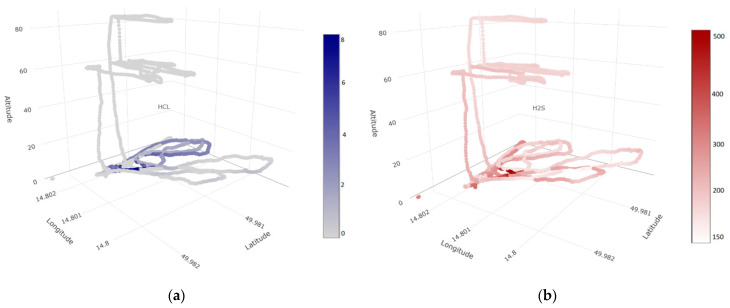
3D models present the most sensitive sensors during data collection: (**a**) HCL sensor and (**b**) H_2_S sensor measured by electronic nose.

The results are promising for further research in this area. Mostly HCL but followed by SO_2_, and H_2_S sensors proved to be the most suitable for chemical substances released when attacked by bark beetles (Figure 7a,b). The other sensors reacted for increased concentrations in the clearing area.

## 4. Discussion

Measuring chemical substances using electronic noses is a promising methodology in forestry [12,21]. This system is already verified for monitoring odors from agricultural farms and dangerous gas leaks from industrial sites [41,42,43,44]. This research provides information on suitable electronic nose sensors for stress detection in the forest ecosystem. Measuring VOCs in the air is challenging in terms of the impossibility of repeatability of measurements under the same conditions. Constantly changing climatic and weather conditions change concentrations and direction of spread [2,3,4].

Our research demonstrated the detectability of a specific substance, which is released during stress in spruce trees, by specific electronic nose sensors. The HCL, H_2_S, and SO_2_ sensor during ground measurements (first and second ground data collection) was the most sensitive for our purposes. Some sensors responded to the presence of another substance, so they showed the opposite trend. At this moment, we are not aware of any work that would clearly and undoubtedly prove which substances are released and in which quantities, and, in particular, which sensors react to them. This study is not globally conclusive, and that further study is needed. The research team of Paczkowski et al. [21] achieved similar results in the detection of bark beetle infestation, where they compared the sensitivity of GGS2330 SnO_3_, GGS1330 SnO_2_, and GGS5330 WO_3_; they recorded the highest reaction with the GGS2330 sensor with a SnO_3_ surface suitable for detecting easily thermally oxidized VOCs.

Early detection of infestation is desirable not only in forestry but also in agriculture. The research methodology remains similar, and the aim of the assessment is the interaction between the insect and the plant. An accuracy of 94.2–99.2% was achieved when monitoring wheat aphid infestation; data collected were obtained by a cheap electronic nose [19]. The signal from the electronic nose was used to distinguish rice infested and not infested by the Nilaparvata lugens pest; the correlation coefficient reached a value of 0.78 between the predicted pests and the actual ones recorded using machine learning models [33].

Ground data collection provides important information, but for forestry applications, it would be better to have comprehensive data from several measurement heights, and thus also above the tree canopy. In this brief report, we have not proven a correlation between the distance from the attacked trees and the values measured by the sensors during UAV data collection. First, we flew at a height of 60 m; then, we climbed vertically to a height of 80 m. This procedure was chosen to minimize the influence of the downwash effect [45]. We used a sampling tube for data collection; the Teflon tube was 1 m long and was used to collect air in front of the UAV. A meter distance is sufficient to minimize disturbances during data collection [46]. Despite these measures, the same results were not achieved as with ground data collection. It is possible that the mixing of wind above the tree canopy caused this. Another factor may be the substance values were below the detection limit of the tested sensors, and measuring with a drone disturbs the air with the movement of the rotors (downwash effect). Therefore, paying attention to this aspect before data measurement is crucial. The effect of the height above the canopy was investigated by a Winsen ME4-C_2_H_4_ electrochemical sensor in a research study focused on the ideal harvest time of apples. The results show that the ethylene concentration decreased by 95% at 4 m and 90% at 2 m above the trees [47].

The potential solution may be creating a canopy height model. Based on the data on tree heights, it would be possible to move closer to the forest crowns and, thus, more accurately capture VOCs from the upper part of the crowns. If even this maximum approach to the trees’ canopy would not help, there is the option of manual flight below the tree canopy.

Several factors, including increased drought and insect pests, can cause stress. So, direct detection of the bark beetle is very complicated; the aggregation pheromone, which is produced by representatives of the bark beetle, is in very low concentrations in the forest stand. Therefore, it might be easier to detect just-released VOCs, which signify an increased stress level in trees [31]. In recent years, stress has mainly been caused by bark beetle overgrowth in coniferous forests in the Czech Republic.

Our research points to the suitability of the HCL, H_2_S, and SO_2_ sensors for stress detection. However, further data collection in diverse areas is desirable. For further research, we recommend verifying UAV data collection in close proximity to tree canopies. It would be appropriate to repeat the experiment over a longer time horizon and monitor temporal changes both in the daily regime and in terms of the rate and development of infestation in the forest stand. For global conclusions, more extensive data collection will be necessary, as other factors (e.g., humidity, temperature, air pressure) may influence the results, and it is in no way possible to catch all in one experiment.

## 5. Conclusions

The presented research describes a potential methodology for the early detection of bark-beetle-caused stress in the early stages of infestation (green attack) and dead trees, which are usually the result of the previous attack. Using electronic noses and similar sensors has been a proven methodology for faster data evaluation in the industry for years. Therefore, applying electronic sensing noses in forestry represents such a potential. The main advantage of this method is fast data processing; primary measurement data can be checked in real-time using the software. Early identification of attacked trees would bring enormous ecological and financial savings. We tested several sensors from an electronic nose to detect dead and infested trees, and the HCL sensor performed most reliably, providing accurate and higher correlation values with the distance to the target. The highest accuracy was achieved during the first and second ground measurements. We confirmed our hypotheses, and as part of the research, we managed to detect with specific sensors the increased presence of a substance that identifies bark beetle infestation (i.e., stressed trees). The results from the data obtained by the UAV do not indicate decreasing trends from the targets, which could be caused by a lower concentration of substances above the tree canopy or an influence of higher wind movement. The results presented here are very promising, but the research needs to be followed up with further experiments to verify the use of other sensors, and in different situations.

## Figures and Tables

**Table 1:** Description of collected data, first ground collection, second ground collection, and UAV data collection.

Data Collection	Sample Number	Sample Category	Mean	Standard Deviation
First ground measurement	1148	VOCs	0.372	0.001
		SO_2_	20.007	7.486
		CO	0.235	0.032
		O_3_ + NO_2_	741.040	227.101
		PM_1.0_	16.541	1.102
		PM_2.5_	22.951	1.740
		PM_10_	25.614	1.774
		CxHy	0.079	0.003
		H_2_S	217.614	53.369
		HCL	1.263	1.693
Second ground measurement	811	VOCs	0.376	0.001
		SO_2_	30.709	8.624
		CO	0.253	0.035
		O_3_ + NO_2_	135.970	23.466
		PM_1.0_	15.260	0.769
		PM_2.5_	20.059	1.363
		PM_10_	22.520	1.513
		CxHy	0.085	0.003
		H_2_S	269.391	54.577
		HCL	1.912	1.832
UAV measurement	815	VOCs	0.376	0.002
		SO_2_	23.378	5.061
		CO	0.211	0.026
		O_3_ + NO_2_	171.156	25.642
		PM_1.0_	15.781	0.890
		PM_2.5_	21.509	1.247
		PM_10_	23.927	1.320
		CxHy	0.085	0.001
		H_2_S	217.501	11.957
		HCL	0	0

**Table 2:** Pearson’s correlation coefficient results of data first and second ground collection for dead trees.

Data Collection	Condition of Trees	Sensor	Pearson’s Correlation Coefficient
First ground measurement	Dead trees	VOCs	0.322
		**SO_2_**	**−0.209**
		CO	0.071
		O_3_ + NO_2_	0.629
		PM_1.0_	0.300
		PM_2.5_	0.284
		PM_10_	0.237
		CxHy	−0.071
		**H_2_S**	**−0.132**
		**HCL**	**−0.456**
Second ground measurement	Dead trees	VOCs	−0.036
		**SO_2_**	**−0.360**
		CO	0.394
		O_3_ + NO_2_	0.108
		PM_1.0_	0.189
		PM_2.5_	0.000
		PM_10_	−0.052
		CxHy	−0.146
		**H_2_S**	**−0.432**
		**HCL**	**−0.378**

**Table 3:** Pearson’s correlation coefficient results of data first and second ground collection for early-stage infestation trees.

Data Collection	Condition of Trees	Sensor	Pearson’s Correlation Coefficient
First ground measurement	Early stage of infestation	VOCs	0.230
		**SO_2_**	**−0.164**
		CO	0.062
		O_3_ + NO_2_	0.648
		PM_1.0_	0.274
		PM_2.5_	0.285
		PM_10_	0.219
		CxHy	−0.076
		**H_2_S**	**−0.100**
		**HCL**	**−0.427**
Second ground measurement	Early stage of infestation	VOCs	0.165
		**SO_2_**	**−0.480**
		CO	0.337
		O_3_ + NO_2_	−0.150
		PM_1.0_	0.231
		PM_2.5_	−0.048
		PM_10_	−0.068
		**CxHy**	**−0.339**
		**H_2_S**	**−0.486**
		HCL	−0.118

**Table 4:** Pearson’s correlation coefficient results of UAV collection for early-stage infestation and dead trees.

Data Collection	Condition of Trees	Sensor	Pearson’s Correlation Coefficient
UAV measurement	Early stage of infestation	VOCs	0.445
		SO_2_	0.415
		CO	0.464
		O_3_ + NO_2_	0.501
		PM_1.0_	−0.020
		PM_2.5_	−0.033
		PM_10_	0.021
		CxHy	0.065
		H_2_S	0.250
		HCL	NA
UAV measurement	Dead trees	VOCs	0.346
		SO_2_	0.294
		CO	0.377
		O_3_ + NO_2_	0.357
		PM_1.0_	−0.006
		PM_2.5_	−0.101
		PM_10_	−0.056
		CxHy	0.140
		H_2_S	0.140
		HCL	NA

**Figure A1:**
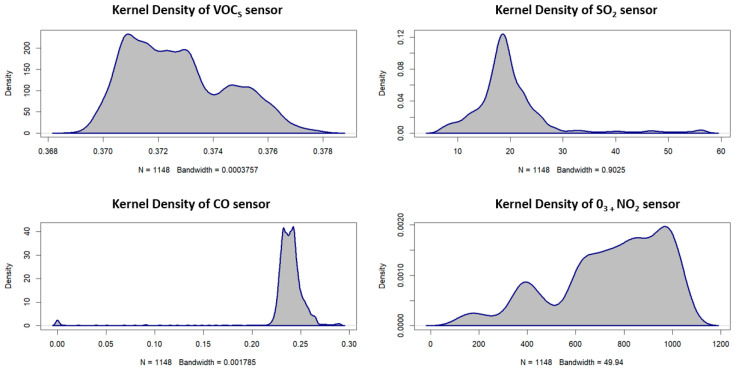
Kernel density for first ground measurement (VOCs sensor, SO_2_ sensor, CO sensor, O_3_ + NO_2_ sensor).

**Figure A2:**
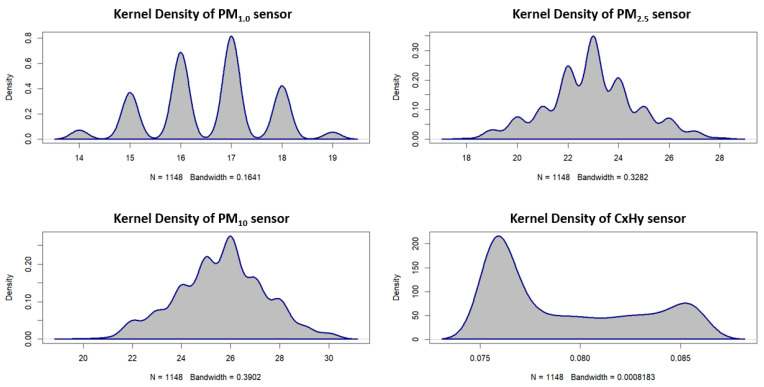
Kernel density for first ground measurement (PM_1.0_ sensor, PM_2.5_ sensor, PM_10_ sensor, C_X_H_Y_ sensor).

**Figure A3:**
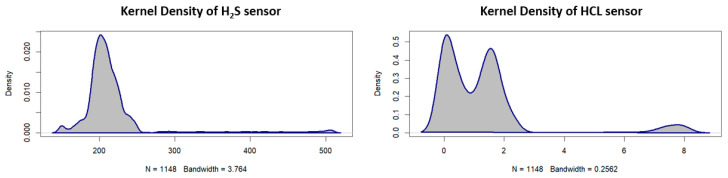
Kernel density for first ground measurement (H_2_S sensor, HCL sensor).

**Figure A4:**
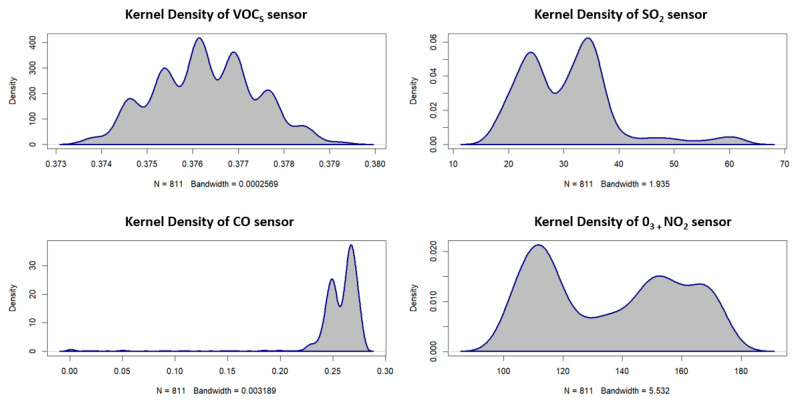
Kernel density for second ground measurement (VOCs sensor, SO_2_ sensor, CO sensor, O_3_ + NO_2_ sensor).

**Figure A5:**
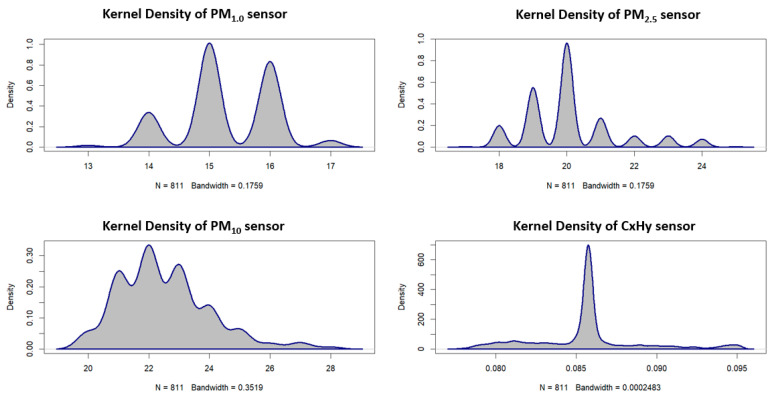
Kernel density for first second measurement (PM_1.0_ sensor, PM_2.5_ sensor, PM_10_ sensor, C_X_H_Y_ sensor).

**Figure A6:**
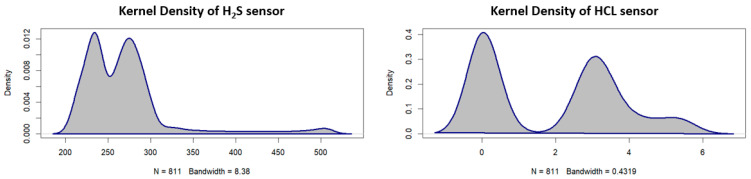
Kernel density for second ground measurement (H_2_S sensor, HCL sensor).

**Figure A7:**
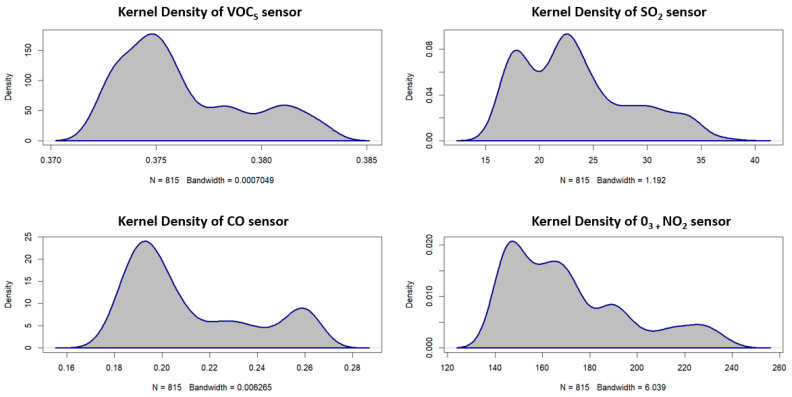
Kernel density for UAV measurement (VOCs sensor, SO_2_ sensor, CO sensor, O_3_ + NO_2_ sensor).

**Figure A8:**
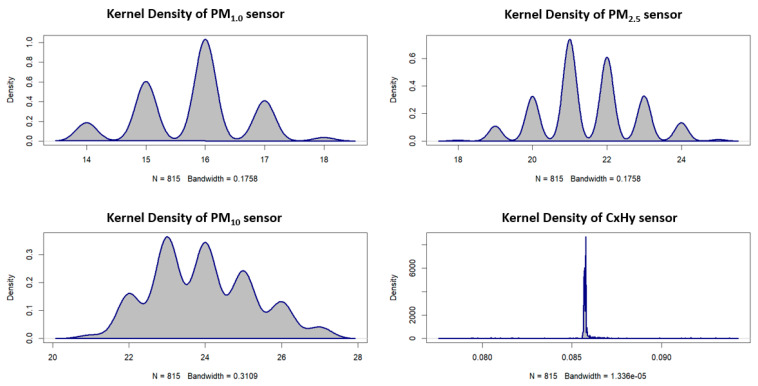
Kernel density for UAV measurement (PM_1.0_ sensor, PM_2.5_ sensor, PM_10_ sensor, C_X_H_Y_ sensor).

**Figure A9:**
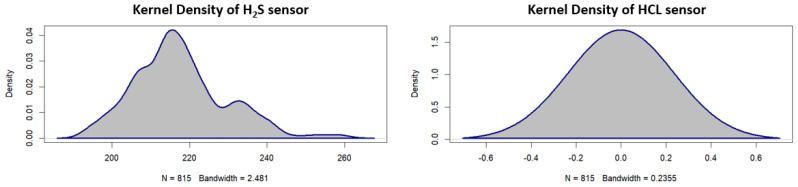
Kernel density for UAV measurement (H_2_S sensor, HCL sensor).

## Data Availability

Data are available upon request by the authors.
